# Quantifying the Size and Duration of a Microburst‐Producing Chorus Region on 5 December 2017

**DOI:** 10.1029/2022GL099655

**Published:** 2022-08-15

**Authors:** S. S. Elliott, A. W. Breneman, C. Colpitts, J. M. Pettit, C. A. Cattell, A. J. Halford, M. Shumko, J. Sample, A. T. Johnson, Y. Miyoshi, Y. Kasahara, C. M. Cully, S. Nakamura, T. Mitani, T. Hori, I. Shinohara, K. Shiokawa, S. Matsuda, M. Connors, M. Ozaki, J. Manninen

**Affiliations:** ^1^ The University of Minnesota Minneapolis MN USA; ^2^ NASA Goddard Space Flight Center Greenbelt MD USA; ^3^ University of Colorado LASP Boulder CO USA; ^4^ Montana State University Bozeman MT USA; ^5^ ISEE Nagoya University Nagoya Japan; ^6^ Kanazawa University Kanazawa Japan; ^7^ University of Calgary Calgary AB Canada; ^8^ ISAS/JAXA Sagamihara Japan; ^9^ Athabasca University Athabasca AB Canada; ^10^ Sodankylä Geophysical Observatory University of Oulu Sodankylä Finland

**Keywords:** microburst precipitation, chorus waves, radiation belt, electron precipitation, wave‐particle interactions

## Abstract

Microbursts are impulsive (<1 s) injections of electrons into the atmosphere, thought to be caused by nonlinear scattering by chorus waves. Although attempts have been made to quantify their contribution to outer belt electron loss, the uncertainty in the overall size and duration of the microburst region is typically large, so that their contribution to outer belt loss is uncertain. We combine datasets that measure chorus waves (Van Allen Probes [RBSP], Arase, ground‐based VLF stations) and microburst (>30 keV) precipitation (FIREBIRD II and AC6 CubeSats, POES) to determine the size of the microburst‐producing chorus source region beginning on 5 December 2017. We estimate that the long‐lasting (∼30 hr) microburst‐producing chorus region extends from 4 to 8 ΔMLT and 2–5 ΔL. We conclude that microbursts likely represent a major loss source of outer radiation belt electrons for this event.

## Key Points

- We use multipoint observations to estimate the size of a long‐lasting microburst‐producing chorus region beginning on 5 December 2017
- We estimate that the microburst‐producing chorus region for this event extends from 4 to 8 ΔMLT and 2–5 ΔL
- Microburst precipitation from this event likely constitutes a major source of electron loss from the outer radiation belt

## 1 Introduction

Many competing processes contribute to the dynamic formation and depletion of the Earth's radiation belts, including radial transport, local wave acceleration, particle loss to the magnetopause, particle precipitation into the atmosphere, and others (see review by Ripoll et al., 2020; Thorne, 2010). These competing mechanisms typically occur simultaneously and are energy dependent; understanding the importance of each is fundamental to radiation belt physics.

Electron microbursts are impulsive (<1 s) injections of energetic (few keV to >MeV) electrons into the atmosphere that may represent a major loss source from the radiation belt during storm main phase and recovery (Millan & Thorne, 2007). Microburst electron precipitation was first observed in the 1960s by balloon measurements of bremsstrahlung X‐rays produced by precipitating electrons as they enter Earth's atmosphere (K. A. Anderson and Milton, 1964; Winckler et al., 1962). Microbursts have been observed in situ on satellites (J. B. Blake et al., 1996; Imhof & Nightingale, 1992), and via X‐rays on balloons (Millan et al., 2002). Despite a long history of observations, we still do not understand the detailed physics of the underlying scattering mechanism nor the importance of microburst precipitation as a loss mechanism for outer belt energetic electrons (L. W. Blum and Breneman, 2020 review; L. Blum et al., 2015; Breneman et al., 2017, Douma et al., 2019). This is in part due to a lack of data coverage during events when microbursts are observed over a large region and extended time. Although microbursts are short‐lived, they can have fluxes more than an order of magnitude higher than the background precipitation (Lorentzen, Blake, et al., 2001). As such, microburst precipitation can potentially be a significant loss mechanism for outer radiation belt electrons.

The inherently bursty nature of whistler mode chorus waves, and their ability to resonate with a wide range of electron energies, make them the primary candidate for the generation of electron microbursts. Surveys have shown similarities in occurrence of microbursts and whistler mode chorus waves, including distributions in L and MLT and variation with magnetic activity level (Douma et al., 2017). Both share similar sub‐second durations and occur at a similar cadence. The evidence to date thus strongly suggests that the dominant cause of microbursts is resonant scattering into the loss cone by chorus (Breneman et al., 2017; Kersten et al., 2011; Lorentzen, Blake, et al., 2001, Lorentzen, Looper, and Blake 2001; Nakamura et al., 2000; Thorne et al., 2005). However, details of the scattering, including scattering magnetic latitude (equatorial or off‐equatorial), and the exact nature of resonance remain unverified or unknown. Recently, Miyoshi et al. (2020) has proposed a model that relativistic electron microbursts are the high‐energy tail of the pulsating aurora. The latitudinal propagating chorus waves cause wide energy electron precipitations from keV energy (pulsating aurora) and MeV energy (relativistic electron microbursts) because the resonance energy depends on the magnetic latitudes. This model is consistent with recent studies that use conjugate observations between ground‐based observations and SAMPEX and FIREBIRD (Kawamura et al., 2021; Shumko et al., 2021; Zhang et al., 2022).

One way to address these questions is with intercomparison of high‐altitude chorus observations made on near‐equatorial satellites and microburst observations on low Earth orbit satellites during magnetic conjunctions. This data set has been realized in recent years with a collaboration from early 2016 to late 2019 between the Electric Fields and Waves (EFW) team on the near‐equatorial Van Allen Probes (RBSP) and the low‐altitude Focused Investigations of Relativistic Electron Burst: Intensity, Range, and Dynamics (FIREBIRD) CubeSats. More than 5,900 min of EFW burst waveform data and >3,300 min of EMFISIS burst waveform data were taken within ±1 hr of ∼813 magnetic conjunctions with FIREBIRD.

This significant conjunction data set has uncovered details of the connection between chorus and microbursts. For example, Breneman et al. (2017) analyzed a close bounce‐loss cone conjunction and found that the chorus‐induced microburst precipitation from 220–985 keV was scattered at off‐equatorial latitudes of ±20–30°. The latitudinal location of the scattering has been found to have a strong effect of the overall scattering rates and energy range (e.g., Shprits et al., 2006; Thorne et al., 2005). Another study (Colpitts et al., 2020) showed the first direct evidence of discrete chorus elements propagating from an equatorial satellite (Van Allen Probe A) to a satellite at higher latitudes (Arase). This study confirmed that these waves can propagate unducted, without selective amplification, to the latitudes necessary to scatter electrons producing microburst precipitation.

The conjunction data set also allows us to estimate the scale size of the scattering region. Breneman et al. (2017) used FIREBIRD data to estimate the differential flux loss rate to the atmosphere due to 220–985 keV microbursts. The time to deplete the entire outer radiation belt through this mechanism can be determined by integrating the estimated average loss rate over the entire chorus source region. The chorus source size has been estimated from transient auroral flashes (Ozaki et al., 2019) and pulsating auroral patches related to isolated chorus elements(Ozaki, Shiokawa, et al., 2018) and the source size can depend on the chorus wave propagation (Ozaki et al., 2021). However, this critical source region size is not well constrained.

An earlier study by B. R. Anderson et al. (2017) also estimated the size of a microburst region using observations from the Balloon Array for Radiation belt Relativistic Electron Losses (BARREL) mission, FIREBIRD, and AC6 and found the region to extend over 5 $R_{E}$ in L shell and 4 hr in MLT. However, the MLT extent was underestimated due to lack of early MLT coverage from any of the datasets, and they did not include observations of chorus waves. Lorentzen, Looper, and Blake (2001) also assumed microbursts occurred over 6 hr in MLT and 2 $R_{E}$ in L shell for their global loss calculation. The overall contribution that microburst precipitation has on outer belt loss is uncertain.

To resolve this open question, we use this new database, augmented with additional satellite‐borne and ground‐based data, to determine the typical size and duration of this region of wave‐particle interaction following a magnetic storm on 5 December 2017.

## 2 Instrumentation and Datasets

This study combines chorus wave measurements from Van Allen Probes (RBSP) (Mauk et al., 2013), Arase (ERG) (Miyoshi, Hori, et al., 2018; Miyoshi, Shinohara, et al., 2018), and ground‐based VLF stations with complimentary low Earth orbit (LEO) microburst measurements from the FIREBIRD II (Klumpar et al., 2015) and Aerospace Corporation AeroCube‐6 (AC6) CubeSat missions, and particle flux measurements from the Polar Operational Environmental Satellites (POES, including the National Oceanic and Atmospheric Administration and Meteorological Operational satellites, NOAA/MetOp) (Evans & Greer, 2004; Rodger et al., 2010). Combined, these data provide information on the extent and duration of microburst‐producing chorus regions.

### 2.1 Chorus Measurements

Chorus wave measurements were obtained from the Van Allen Probes (RBSP A, B), Arase, and ground‐based VLF data from the Canadian Space Agency's Geospace Observatory Array for Broadband Observations of VLF/ELF Emissions (GO‐ABOVE) receivers (Cully et al., 2014), PWING (study of dynamical variation of Particles and Waves in the INner magnetosphere using Ground‐based network observations) and Sodankylä networks (Shiokawa et al., 2017) (a detailed description of the Kannuslehto receiver is given in Manninen, 2005). RBSP had a near‐equatorial elliptical orbit ranging from ∼600 km to >6 $R_{E}$ apogee. Due to the 11° magnetic inclination, magnetic latitudes of up to 20° and *L* values of up to *L* > 6 were sampled. The Arase satellite has an elliptical orbit, with apogee at 32,000 km, perigee at 400 km, and inclination of 31° magnetic latitude (Miyoshi, Shinohara, et al., 2018).

Wave data were provided by the RBSP Electric Field and Waves (EFW) (Wygant et al., 2013) and the Electric and Magnetic Field Instrument Suite and Integrated Science (EMFISIS) (Kletzing et al., 2013) instruments, and energetic electron data were provided by the Magnetic Electron Ion Spectrometer (MagEIS) (J. Blake et al., 2013). Chorus waves on Arase are identified from one second resolution spectra from Arase's Onboard Frequency Analyzer (OFA); part of the Plasma Wave Experiment (PWE) instrument suite (Kasaba et al., 2017; Kasahara et al., 2018; Matsuda et al., 2018; Ozaki, Yagitani, et al., 2018). Energetic electron data was obtained by the High‐energy electron experiments (HEP) (Mitani et al., 2018) onboard Arase.

### 2.2 Microburst Measurements

Electron microburst measurements were obtained from FIREBIRD II and AC6‐A CubeSats. FIREBIRD II was launched in 2015 into an orbit of 632 km by 433 km at 99.1° inclination. The twin FIREBIRD CubeSats, Flight Units 3 and 4 (FU 3 and FU 4 hereafter), detect energetic electrons from ∼250–1,000 keV in five differential energy channels and one integral (>1 MeV) channel (Crew et al., 2016; Klumpar et al., 2015; Spence et al., 2012). For microburst observations on FIREBIRD, we use high resolution data (50 msec sampling) from the lowest differential energy channel of each collimated detector (230–300 keV on FU3 and 219–283 keV on FU4; Johnson et al., 2020). Microbursts are often observed also in the higher energy channels but generally at decreased fluxes. AC6‐A was launched in 2014 into a low Earth orbit of 620 km by 700 km at 98° inclination, carrying a suite of three radiation dosimeters. The current study uses the >35 keV dosimeter for electrons, which operates in a 10 Hz mode to resolve microbursts. To identify microbursts on both FIREBIRD and AC6‐A we combined an automated detection method (O’Brien et al., 2003; Shumko et al., 2020) with a visual verification.

### 2.3 Particle Flux Measurements

Electron flux measurements from the Medium Energy Proton and Electron Detector (MEPED) instruments on the POES satellites (NOAA15, NOAA18, NOAA19, METOP01, and METOP02) provide a measure of strong electron flux in the bounce loss cone (BLC). This flux is expected to increase in regions where strong microburst precipitation is occurring. While the 2‐s cadence of these measurements cannot resolve microbursts, their excellent spatial coverage is invaluable for identifying the likely size of the precipitation region over time. It should be noted that POES has limited spatial coverage between 10 and 15 MLT (see Figure S1 in Supporting Information S1) in years after the MEPED instrument aboard NOAA16 was no longer in operation (2005). The POES Space Environment Monitor (SEM/2) MEPED data used in this study uses processing as described by Pettit et al. (2021) and is referred to as the MEPED Precipitating Electron (MPE) data set. The MPE data set calculates differential flux using all three POES electron channels as well as the P6 channel, which can be used as a proxy for relativistic electrons (Yando et al., 2011). The resulting flux has an energy range of 27 keV–8.9 MeV with 27 differential energy levels. Using the differential electron flux, precipitating flux is estimated using both detectors aboard POES, giving BLC flux estimates at all the POES satellite locations. A flux versus energy threshold profile for the satellites was developed, which was then compared to the estimated BLC fluxes for energies between 84.7 and 320.8 keV (energies commonly scattered by chorus, e.g., Li et al., 2013; Drozdov et al., 2020; Chen et al., 2022 and references therein). The threshold profile was generated by filtering all the MPE spectral data from 2014 to 2020 using a threshold daily Kp‐index of 5 or greater. From the filtered data a median was calculated for each energy level available in the MPE spectral data set. We then applied the threshold on the days of interest by removing all BLC flux that did not meet or exceed the threshold median. A median was chosen in lieu of a mean since BLC flux does not follow a Gaussian distribution. Since the MPE data set measures all precipitating flux at all the POES measurement locations on a given day, the purpose of this analysis was to remove lower levels of BLC flux in an attempt to isolate precipitation caused by chorus waves. However, precipitation caused by other means (EMIC waves, etc.) may still show up in the results.

## 3 Case Study Observations and Results

This study uses simultaneous chorus, microburst, and electron flux observations made from 5 December 2017 00:00 UT to 6 December 2017 06:00 UT. Geomagnetic activity prior to December 5th was relatively low until a weak (Dst ∼ −45 nT) storm late on December 4th. This was accompanied by enhanced electron fluxes, as observed on MagEIS, in association with substorm activity (peaks in AE from 600 to 900 nT), which continued for several days. After December 6th, there was a gradual recovery, with no storms for the next few days.

There were four magnetic conjunction events (with RBSP and FIREBIRD within 1 MLT and 1 L shell of each other on 5 December 2017) during which FIREBIRD obtained high resolution data. Since we focus on the overall extent of the microburst‐producing chorus source region, we include observations taken throughout the interval of interest. RBSP and Arase made multiple passes through the dawn‐to‐dusk MLT sectors across noon. FIREBIRD and AC6‐A also made multiple microburst observations in these same regions. All data sets use L shells computed using the Internal Geomagnetic Reference Field (IGRF) internal model (Thébault et al., 2015) and the Tsyganenko (1989) (T89) external model (Tsyganenko, 1989), with POES using the IGRF model. Because POES data is binned, the conclusions are not affected by small differences due to the model.

### 3.1 Chorus Wave Observations

Starting near the beginning of the 5 December 2017 event, RBSP, Arase, and ground‐based VLF stations observe >30 hr of near‐continuous lower‐band chorus activity, with enhancements associated with the substorm injections. We focus on three nine‐hour periods, selected to provide sufficient spatial coverage of the inner magnetosphere outside of the plasmasphere while still allowing insight into the overall time evolution of the microburst producing region. Wave observations during the first period (∼02:00–11:00 UT on 5 December 2017) were made primarily by Arase, with the strongest waves from ∼03:20 to 06:30 UT. Waves during the second period (∼12:00–21:00 UT on 5 December 2017) were mainly observed by RBSP, with the strongest from ∼11:20 to 18:10 UT (∼14:20–20:42 UT) on RBSP‐A (RBSP‐B). The third period (∼21:00 UT on 5 December 2017 to 06:00 UT on 6 December 2017) consisted of weak chorus observed by both RBSP and a separate chorus region observed by Arase.

Throughout the entire period, electric and magnetic wave amplitudes of >15 mV/m and >95 pT were measured on RBSP. Chorus wave normal angles (the angle of the wave vector with the background magnetic field) at low magnetic latitudes (near the geomagnetic equator) as observed on RBSP and Arase were mostly quasi field aligned (<45°) with a mix of quasi‐perpendicular (>45°) for weaker waves. At higher magnetic latitudes of 25°–35°, where the chorus waves likely create relativistic microbursts, wave normal angles observed on Arase varied from quasi‐parallel to quasi‐perpendicular.

**Figure 1:**
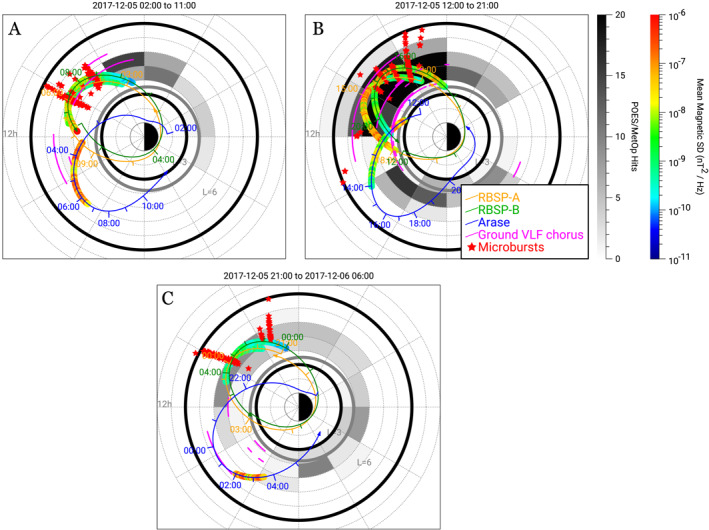
9‐hr plots from 02:00 to 11:00 UT (a) on 5 December 2017, 12:00 to 21:00 UT (b) on 5 December 2017, and 21:00 UT on 5 December 2017 to 06:00 UT on 6 December 2017 (c) showing multi‐instrument observations. Dial plots show the MLT and L shell locations of RBSP A and B (orange and green lines respectively) and Arase (dark blue line). The rainbow color bar shows the mean magnetic spectral density of the chorus waves (between 0.1 and 0.5 Fce) observed on RBSP and Arase. The magenta lines represent the observed chorus from ground‐based VLF stations. The gray color bar indicates regions where POES observed strong electron precipitation (“strong” meaning the measured BLC flux exceeds the threshold profile as described above–a “hit” is a measurement that exceeds our threshold within a bin), and the red stars indicate microburst observations from Focused Investigations of Relativistic Electron Burst: Intensity, Range, and Dynamics and/or AC6‐A. The inner and outer black circles in each plot show L shells of three and eight respectively. The gray ovals show the plasmapause location based on the Moldwin et al. (2002) Kp model.

Figure 1 shows multipoint observations of the L/MLT distribution of lower‐band chorus waves, microburst signatures, and strong electron precipitation. Because the plots each include 9 hr of data, a magnetic conjunction cannot be implied based solely on overlapping tracks. Care should also be taken when analyzing the strength of chorus waves, particularly on Arase, because there are other waves in the same frequency band which may not be chorus (see Figure S2 in Supporting Information S1). Chorus waves initiate around MLT of 6 for the first two time periods and around 7 MLT for the later period, however, due to limited early MLT coverage, these cutoffs are not exact, and we are therefore potentially underestimating the size of the chorus region. At later MLTs however, Arase observed chorus wave cutoffs around 15.5 MLT for the 02:00 to 11:00 UT period and 14 MLT for the 12:00 to 21:00 UT period. Because Arase coverage extends beyond these MLTs, these boundaries better reflect the later MLT extent of the region. For the 12:00 to 06:00 UT on 6 December 2017 period, chorus waves are not observed past 11 MLT from RBSP. Chorus waves were observed by Arase between 15 and ∼16 MLT. It should be noted however that there is some indication from the VLF ground stations that there may be chorus in between the observations from RBSP and Arase (see Figure 1).

### 3.2 Microburst/Precipitation Observations

Microbursts are observed on both FIREBIRD CubeSats (FU3 and FU4) and on AC6‐A. Both missions provide good radial coverage of the microburst region but have limited MLT coverage. Therefore, as described above, we supplement these observations with POES electron precipitation (BLC fluxes between 84.7 and 320.8 keV).

For the first 9‐hr period (Figure 1a), FU3, FU4, and AC6‐A made measurements between ∼8 and 11 MLT and ∼20 to 23 MLT and POES pass through most MLTs (with limited coverage between 10 and 15 MLT, see Figure S1 in Supporting Information S1). We observe a strong electron precipitation region beginning around 4 MLT, extending from approximately 4 to 5 L. This region continues until about 8 MLT, with additional microburst observations from FIREBIRD and AC6‐A between ∼9 to 10 MLT from 5 to 8 L. There are no microburst observations past this MLT due to observational limits (i.e., there are two additional AC6‐A passes through 11 MLT, but no FIREBIRD and limited POES passes), but there is strong electron precipitation observed just past 12 MLT. It should be noted that POES does observe electron precipitation in the 10 to 12 MLT region (see Figure S3 in Supporting Information S1), but these observations do not exceed our threshold profile and are therefore not counted as strong electron precipitation. The observations in these regions are also very limited (see Figure S1 in Supporting Information S1).

For the second 9‐hr period (Figure 1b), FU3, FU4, and AC6‐A made measurements between ∼6 and 14 MLT and ∼16–24 MLT and POES sampled many MLT regions (again, with limited coverage between 10 and 15 MLT). A large (4–7 L) precipitation region (observed by POES) initiates around 6 MLT that continues until 12 MLT (note that a smaller region also occurs from 2 to 6 MLT). There are microburst observations extending from 7 to 14 MLT, extending from 4.5 to 8 L. Another region of precipitation is observed between 14 and 22 MLT around 3–6 L, but no chorus is observed even though Arase took observations throughout this region. Therefore, it is likely that this dusk precipitation region is caused by other waves such as EMIC waves (e.g., Capannolo et al., 2021). It should be noted that Arase observed He + ‐band EMIC waves from ∼15:30 to 15:45 UT. During this time no chorus waves were detected. Extensive work has been done using POES data to study EMIC‐driven precipitation (e.g., Carson et al., 2013; Rodger et al., 2015; Hendry et al., 2016, 2019; Capannolo et al., 2021). Precipitation bands have also been observed in lieu of microbursts to indicate EMIC‐produced precipitation events (L. Blum et al., 2015), but investigation of these is beyond the scope of the current study.

For the third 9‐hr period (Figure 1c), FU3, FU4, and Ac6‐A made measurement between ∼6 and 14 MLT and ∼16–24 MLT and POES observed at most MLTs. Precipitation on POES is observed within most MLT regions, and microbursts are observed from ∼5–8 L between 7 and 10 MLT.

### 3.3 Multipoint Observations and Results

**Figure 2:**
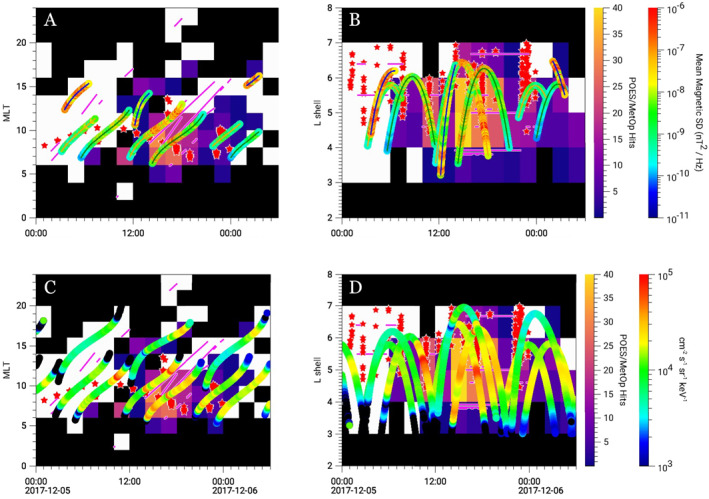
(a) MLT versus UT plot showing observations of chorus (rainbow color bar, representing the mean magnetic spectral density of lower‐band chorus observed on RBSP and Arase), magenta lines showing positive detections of chorus from VLF ground stations, microbursts (red stars, representing microbursts observed by Focused Investigations of Relativistic Electron Burst: Intensity, Range, and Dynamics and AC6‐A), and strong electron precipitation (purple/orange color bar, representing electron precipitation observed by POES). Data is only plotted for regions where there was coverage of both chorus and precipitation. Therefore, the black regions represent areas where there is only coverage of precipitation, or no coverage at all. (b) L shell versus UT plot showing the same observations. (c) MLT versus UT plot showing electron fluxes (rainbow color bar) from MagEIS (RBSP FESA spin‐averaged differential electron flux for 183 keV electrons) and HEP (Arase FEDO omni‐directional electron flux for 176.7 keV electrons). (d) L shell versus UT plot for electron fluxes from MagEIS (RBSP) and HEP (Arase).

Figures 2a and 2b show the same observations as Figure 1 but plots the MLT and L data separately versus time for the entire event study (from 5 December 2017 00:00 UT to 6 December 2017 06:00 UT). Figures 2c and 2d show the measured electron fluxes from MagEIS and HEP. Data are only plotted for regions where simultaneous observations of chorus and microbursts/precipitation could have been made (i.e., the black regions have no coverage from RBSP and/or Arase). Figures 2a and 2b demonstrate the consistency in the size (MLT and L) of the microburst and chorus regions throughout the day. Figures 2c and 2d show that the locations of strong electron precipitation and increased microburst flux coincide with regions of high electron fluxes (measured by RBSP and Arase). For example, from 14:00 to 20:00 UT, the electron fluxes increase, and FIREBIRD and AC6‐A observe a number of microbursts. Another region of high electron and microburst/precipitation fluxes occurs between 10:00 and 12:00 UT. Both of these regions also have strong electron precipitation observed by POES. MagEIS/HEP electron flux is also lower earlier in the day, which is likely contributing to the lack of strong electron precipitation observed by POES. POES does observe precipitation in these regions (see Figures S3 and S4 in Supporting Information S1), but it is not strong enough to exceed our threshold profile. The RBSP‐A and FU4 conjunction observations indicate that the microburst flux was considerably higher at 23:00 UT than 16:30 UT even though average chorus wave amplitudes were larger at the earlier time. Determining the reason for this discrepancy is beyond the scope of this study.

**Figure 3:**
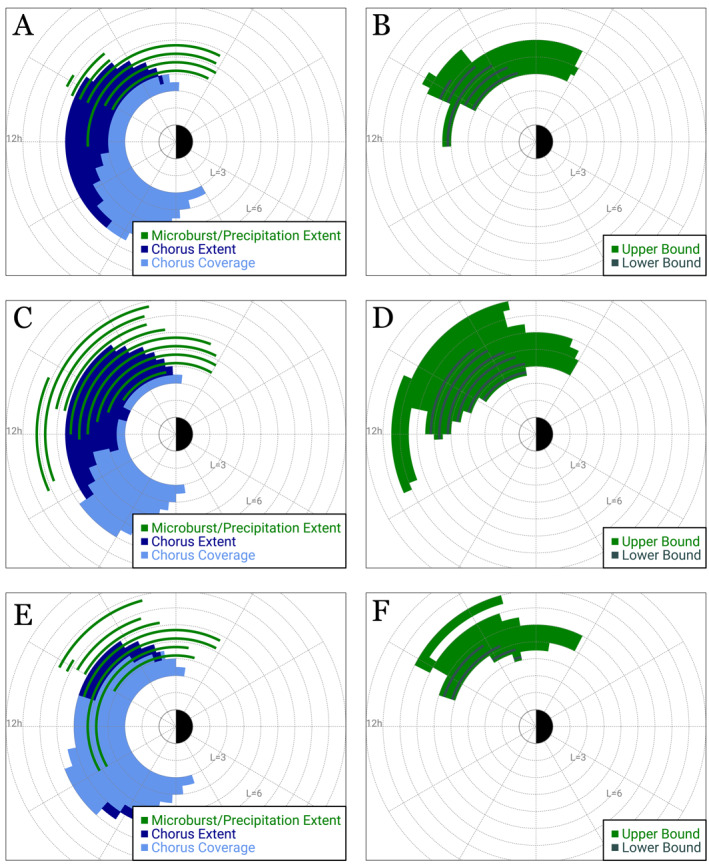
(a, c, e) Overview of three 9‐hr periods of persistent chorus and precipitation observations. Green bars show the microburst/precipitation extent between 4 and 14 MLT. Dark blue bars show the chorus extent. Light blue bars show the chorus coverage from RBSP and Arase. (b, d, f) Upper and lower bounds on the size of the microburst‐producing chorus region for the three periods. Gray bars show the lower bounds of the region (i.e., the overlap between chorus and microburst/precipitation observations). Green bars show the upper bounds of the region (i.e., regions where chorus and microburst/precipitation are observed and regions where only microburst/precipitation is observed but no chorus waves due to lack of coverage).

To constrain the region size we analyze the MLT and L extent of chorus and precipitation/microbursts for the three 9‐hr periods discussed above. Figures 3a–3e present the chorus and microburst/precipitation extent for these intervals and show clear overlap between the chorus wave and microburst/precipitation observations. There are other regions where microburst/precipitation is observed; however, there was no spacecraft coverage to detect chorus waves. Figures 3b–3f show the upper/lower bounds on the size of the microburst‐producing chorus regions for these three time periods. We find that the microburst‐producing chorus region ranges from 4 to 8 ΔMLT and 2–5 ΔL.

It should be noted that due to there being more than one large precipitation region (e.g., the three precipitation regions during the 12:00–21:00 UT time period in Figure 1b described above) we must put boundaries on our calculations of the region size to exclude precipitation regions that are likely not caused by chorus wave interactions. Therefore, the green regions in Figure 3 show the MLT extent of the precipitation between 4 and 14 MLT, excluding the precipitation region observed from 14 to 22 MLT that is likely caused by other sources. It should also be noted that assumptions must be made about the continuity of chorus waves between two observational points. Therefore, we assume chorus waves are active throughout the region if observations show chorus wave activity when the satellites are in the inner magnetosphere outside of the plasmasphere.

These three long‐lived (∼9 hr) events provide the first (lower bound) estimates for the microburst‐producing chorus region. Note that all three periods had limited chorus coverage prior to 6 MLT and beyond 6 L, suggesting we may be underestimating the extent of the chorus region.

## 4 Discussion and Conclusions

Constraining the extent of the microburst‐producing chorus region is vital to understanding whether microburst precipitation is a significant loss source for relativistic outer radiation belt electrons (Breneman et al., 2017). We have presented an event study for a 30‐hr period (00:00 UT on 5 December 2017 to 06:00 UT on 6 December 2017) where simultaneous chorus wave (from RBSP, Arase, and ground‐based VLF stations), microburst (from FIREBIRD and AC6‐A), and electron precipitation observations (from POES) were made.

Chorus wave normal angles varied from quasi‐parallel at low magnetic latitudes to quasi‐perpendicular at higher magnetic latitudes. These observations are consistent with Colpitts et al. (2020) who compared RBSP and Arase observations of the same chorus wave packets, along with a ray tracing analysis, to verify unducted propagation from the near‐equatorial source to higher latitudes. Additional studies have shown both ducted (Chen et al., 2020) and nonducted (Chen et al., 2021; Ozaki et al., 2021) propagation can occur. Our observations support the theory that near equatorial (RBSP) chorus propagates to off‐equatorial (Arase) latitudes, where the chorus waves become more oblique and can resonate with hundreds of keV electrons to produce microbursts.

We estimate that the microburst‐producing chorus region extends from 4 to 8 ΔMLT and 2–5 ΔL. Both our lower bound of the size of the region (ΔMLT of 4 and ΔL of 2) and our upper bound (ΔMLT of 8 and ΔL of 5) are larger than the bounds (ΔMLT from 1 to 5 hr) found by Breneman et al. (2017). Using our extents and the microburst flux assumption of Breneman et al. (2017), we find the loss timescales range from 8 to 12 hr, suggesting microburst precipitation is likely a major loss source of outer radiation belt electrons for this event. However, because there were multiple injections throughout the day, comparing the calculated loss rate to other loss processes becomes difficult. We conclude, as did Breneman et al. (2017), that microburst precipitation constitutes a major source of electron loss from the outer radiation belt.

To further constrain the importance of microburst loss, we plan to perform a statistical study of all days with magnetic conjunction events between RBSP and FIREBIRD, incorporating additional satellite‐borne and ground‐based data when available, similar to the study presented here. Including all events will help to constrain the size of the region through improved spacecraft coverage and determine if constraints depend on geomagnetic activity.

## Supporting information

Supporting Information S1

## Data Availability

Science data of the Van Allen Probes (RBSP) used in this study can be found on the Coordinated Data Analysis Web (CDAWeb) (https://cdaweb.gsfc.nasa.gov/) (EFW data is also available on http://www.space.umn.edu/rbspefw-data/ and EMFISIS on https://emfisis.physics.uiowa.edu/data/index). The FIREBIRD II data are available at http://solar.physics.montana.edu/FIREBIRD_II/ and the AC6 data are available at https://rbspgway.jhuapl.edu/ac6. Science data of the ERG (Arase) satellite were obtained from the ERG Science Center operated by ISAS/JAXA and ISEE/Nagoya University (https://ergsc.isee.nagoya-u.ac.jp/index.shtml.en, Miyoshi, Hori et al., 2018). The present study analyzed PWE OFA L2 power spectrum data v02_02 (https://doi.org/10.34515/DATA.ERG-08001), PWE WFC L2 electric field spectrum data v00_02 (https://doi.org/10.34515/DATA.ERG-09002), PWE WFC L2 magnetic field spectrum data v00_02 (https://doi.org/10.34515/DATA.ERG-09003), HEP L2 omniflux data v03_01 (https://doi.org/10.34515/DATA.ERG-01001), MGF L2 8 s spin‐averaged data v03.04 (https://doi.org/10.34515/DATA.ERG-06001), and Orbit L2 v03 data v03 (https://doi.org/10.34515/DATA.ERG-12000). Sodankylä Geophysical Observatory is located 120 km north of the Arctic Circle in Finland and is an independent department of the University of Oulu, with data available at https://www.sgo.fi/Data/archive.php. PWING data is available at https://stdb2.isee.nagoya-u.ac.jp/vlf/. ABOVE is a joint Canada Foundation for Innovation and Canadian Space Agency project developed by the University of Calgary, with data available at https://data.phys.ucalgary.ca/sort_by_instrument/vlf/GO-Canada_ABOVE/. The POES MEPED data is available at the National Centers for Environmental Information (NCEI) at https://www.ngdc.noaa.gov/stp/satellite/poes/dataaccess.html.
