# Supplementary material for: Quantifying the Size and Duration of a Microburst‐Producing Chorus Region on 5 December 2017
Source: Geophys Res Lett. 2022 Aug 15;49(15):e2022GL099655. doi: 10.1029/2022GL099655 (PMC9540649; doi:10.1029/2022GL099655)
Supplement: Supplementary file 1 — Supporting Information S1 [file GRL-49-e2022GL099655-s001.docx]

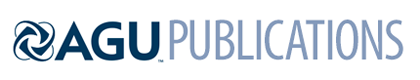


*Geophysical Research Letters*

Supporting Information for

**Quantifying the size and duration of a microburst-producing chorus region on 5 December 2017**

S. S. Elliott^1^, A. W. Breneman^2^, C. Colpitts^1^, J. M. Pettit^3^, C. A. Cattell^1^, A. J. Halford^2^, M. Shumko^2^, J. Sample^4^, A. T. Johnson^4^, Y. Miyoshi^5^, Y. Kasahara^6^, C. M. Cully^7^, S. Nakamura^5^, T. Mitani^8^, T. Hori^5^, I. Shinohara^8^, K. Shiokawa^5^, S. Matsuda^6^, M. Connors^9^, M. Ozaki^6^, J. Manninen^10^

1. The University of Minnesota, MN, USA;
2. NASA Goddard Space Flight Center, MD, USA;
3. University of Colorado LASP, CO, USA;
4. Montana State University, MT, USA;
5. ISEE, Nagoya University, Japan;
6. Kanazawa University, Japan;
7. University of Calgary, AB, Canada
8. ISAS/JAXA, Japan
9. Athabasca University, Canada
10. Sodankylä Geophysical Observatory, University of Oulu, Sodankylä, Finland

**Contents of this file**

Figures S1 to S4

**Introduction**

We present figures showing the locations for the Medium Energy Proton and Electron Detector (MEPED) instruments aboard the Polar Orbiting Environmental Satellites (POES), example chorus wave observations from the Van Allen Probes (RBSP), Arase, and the Canadian Space Agency’s Geospace Observatory Array for Broadband Observations of VLF/ELF Emissions (GO-ABOVE) receivers, and Bounce Loss Cone (BLC) flux calculated by the Precipitating Electron (MPE) data set.


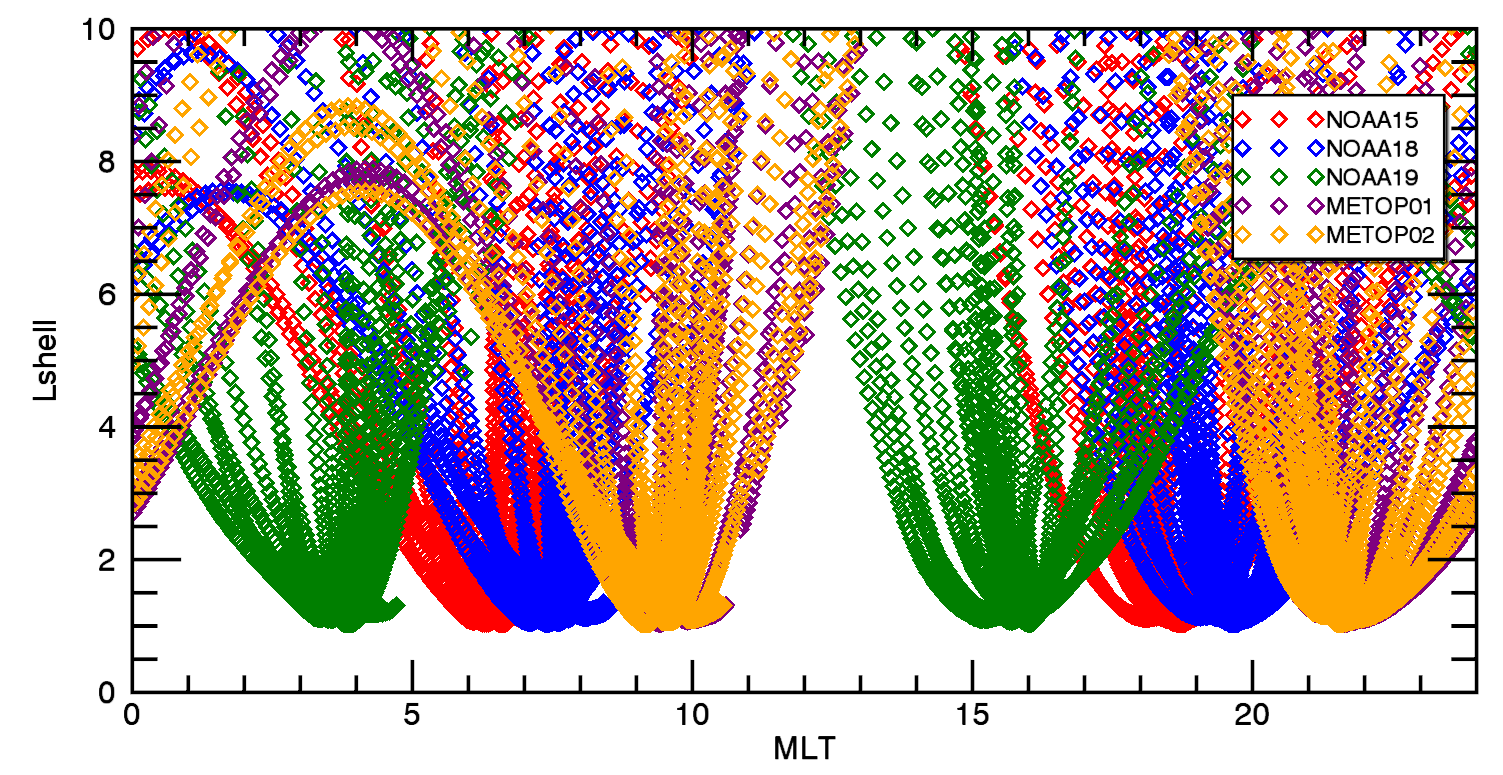
**Figure S1.** Measurement locations for the MEPED instruments aboard the POES satellites are shown as a function of MLT and L-shell for an arbitrary day. The different colors indicate the various satellites as shown in the legend. While only data from a single day is shown, the locations are representative of all time periods. The gap at 12 MLT highlights the missing coverage explained in the text. This area was covered by the NOAA16 satellite prior to its ending of operating in 2005.

Figure S2. Example chorus wave observations for three time periods on 5 December 2017 from RBSP-B (left), Arase (right), and GO-ABOVE Fort Smith (bottom, observed from about 16:00 – 23:00 UT). At times, Arase detects chorus waves along with a structureless hiss-like spectrum (example shown here). For the GO-ABOVE data, unfortunately there was interference in the middle of the event (interference from about 18:10 – 21:00 UT), but the chorus is still distinguishable. For more details on chorus detection by ABOVE observations see *Shen et al.* [2021] and references therein.


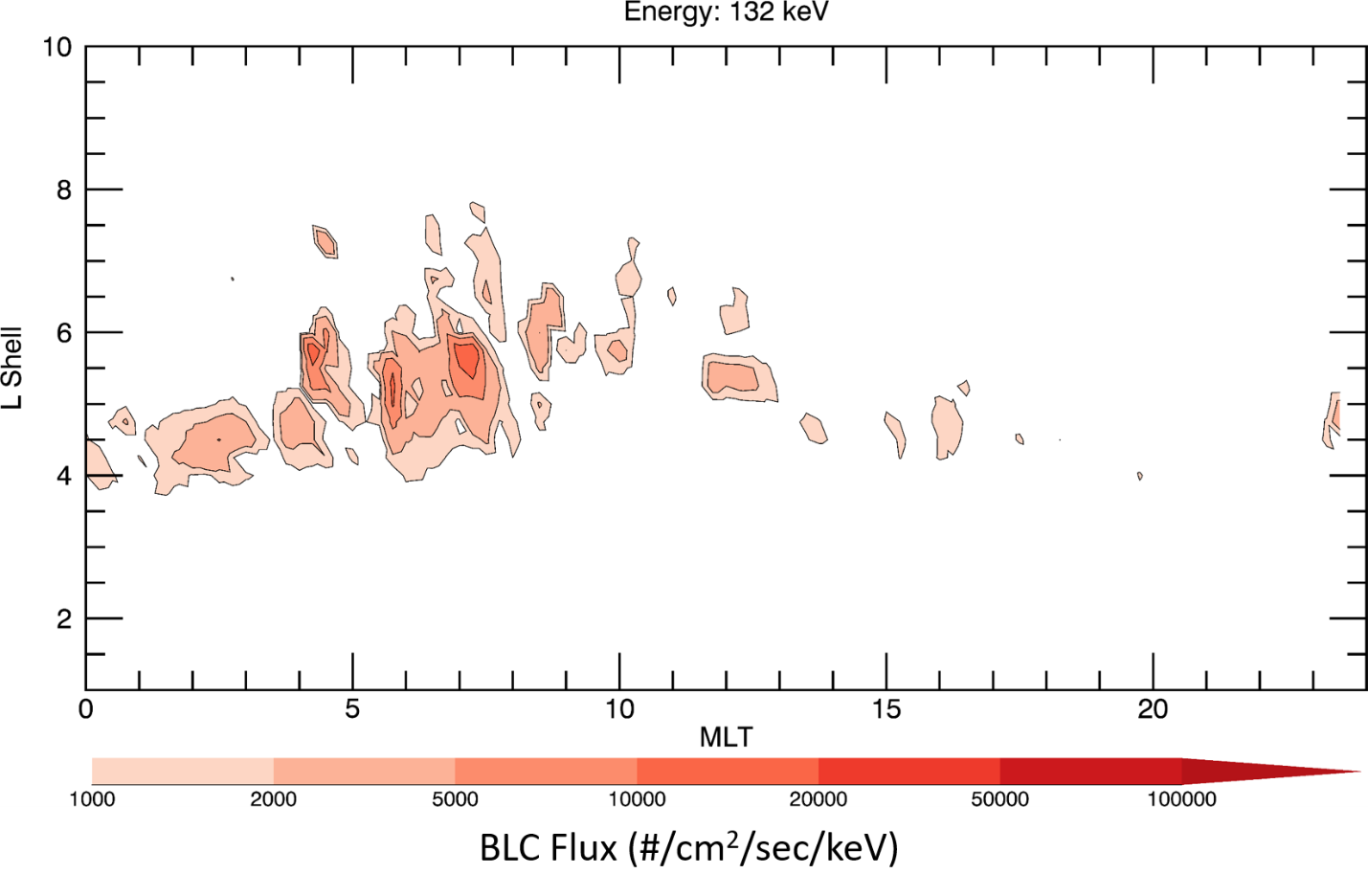


**Figure S3.** Bounce loss cone flux as calculated by the MPE data set at an energy of 132 keV as a function of MLT and L-shell. The time period shown is 0 UTC – 12 UTC on 5 December 2017.


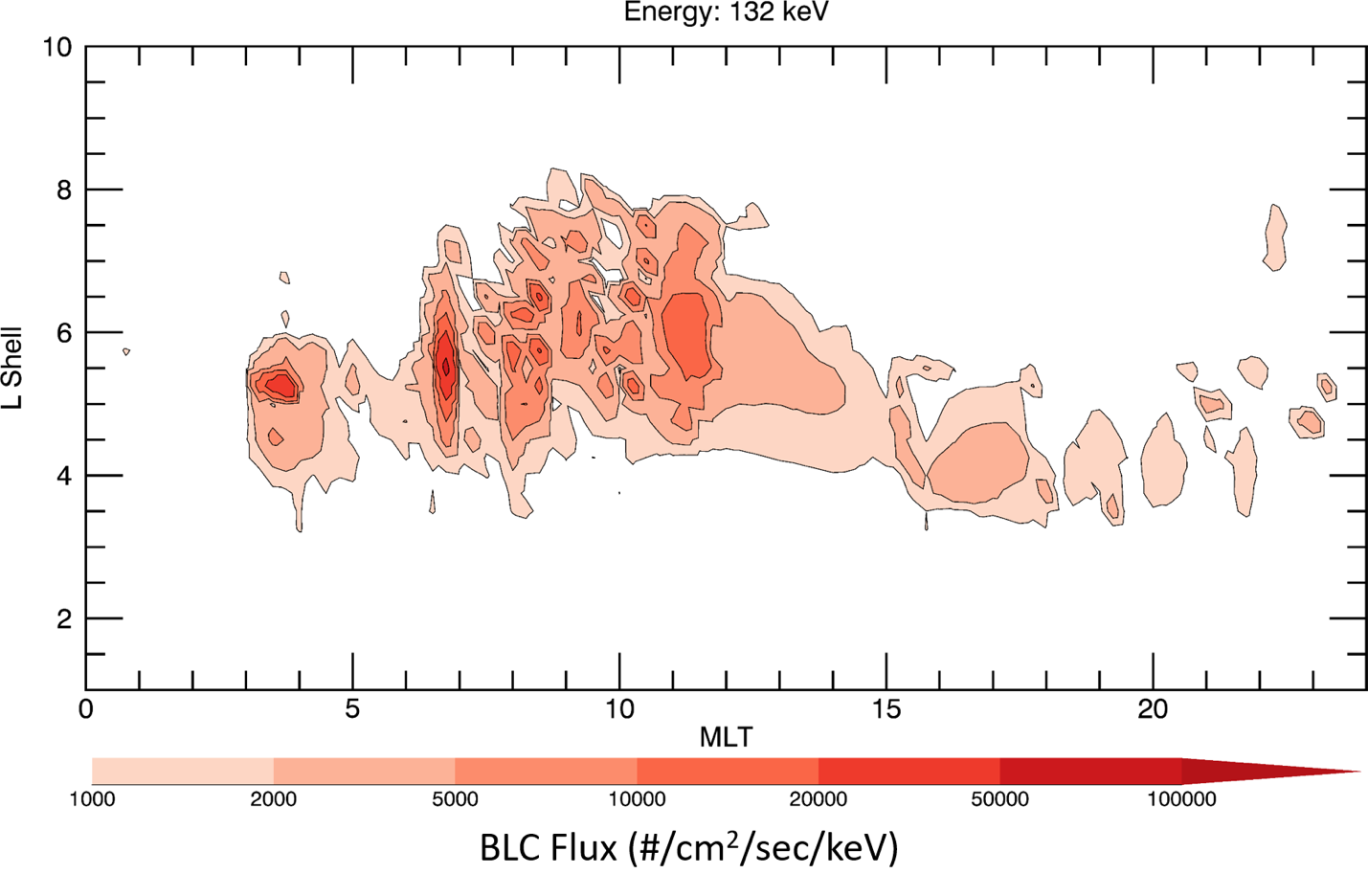


**Figure S4.** Bounce loss cone flux as calculated by the MPE data set at an energy of 132 keV as a function of MLT and L-shell. The time period shown is 12 UTC – 24 UTC on 5 December 2017
